# Controlled biocide release from hierarchically-structured biogenic silica: surface chemistry to tune release rate and responsiveness

**DOI:** 10.1038/s41598-018-23921-2

**Published:** 2018-04-03

**Authors:** Bruno D. Mattos, Blaise L. Tardy, Mohammadi Pezhman, Tero Kämäräinen, Markus Linder, Wido H. Schreiner, Washington L. E. Magalhães, Orlando J. Rojas

**Affiliations:** 1 Department of Bioproducts and Biosystems, School of Chemical Engineering, Aalto University, FI-00076 Aalto, Finland; 2 Integrated Program in Engineering & Materials Science, Federal University of Paraná, Polytechnic Center, Curitiba, 81531-990 Brazil; 3 Laboratório de Superfícies e Interfaces, Universidade Federal do Paraná, Curitiba, 81531-980 Brazil; 4 Embrapa Florestas, Estrada da Ribeira, Km 111, Colombo, 83411-000 Brazil; 5 Department of Applied Physics, School of Science, Aalto University, FI-00076 Aalto, Finland

## Abstract

Biocides are essential for crop protection, packaging and several other biosystem applications. Therein, properties such as tailored and controlled release are paramount in the development of sustainable biocide delivery systems. We explore the self-similar nano-organized architecture of biogenic silica particles to achieve high biocide payload. The high surface area accessibility of the carrier allowed us to develop an efficient, low energy loading strategy, reaching significant dynamic loadings of up to 100 mg·g^−1^. The release rate and responsiveness were tuned by manipulating the interfaces, using either the native hydroxyl surfaces of the carrier or systems modified with amines or carboxylic acids in high density. We thoroughly evaluated the impact of the carrier-biocide interactions on the release rate as a function of pH, ionic strength and temperature. The amine and carboxyl functionalization strategy led to three-fold decrease in the release rate, while higher responsiveness against important agro-industrial variables. Key to our discoveries, nanostructuring thymol in the biogenic silica endowed systems with controlled, responsive release promoting remarkable, high and localized biocidal activity. The interfacial factors affecting related delivery were elucidated for an increased and localized biocidal activity, bringing a new light for the development of controlled release systems from porous materials.

## Introduction

Biocides are essential in agro-industrial applications to improve the yields in crop production and to extend the active life of biodegradable compositions^1,2^. Traditional methods of biocide-based protection often require high initial dosages or repeated applications; consequentially, they result in uncontrolled delivery and short protection time span. Additionally, this leads to excessive, potentially harmful, leaching of the biocide into soil and water. Their bioaccumulation brings hazardous consequences for human health, usually not shown but in the long term. Therefore, it is becoming paramount the development of biocide delivery systems (BDS) that offer controlled or triggered release. However, the development of new, effective, governmentally-approved and safe biocide molecules is a current and expensive challenge^3^. A current paradigm is the development of BDS that minimize the concentration of toxins in soil, food and water while promoting long-term biocidal protection^2^. In this regard, we hypothesize that nanostructured BDS are a promising approach to achieve sustainability, high efficiency and high payload. Thus, engineering BDS at the nanometric scale is an effective answer to this need that requires control of the biocide-carrier interface interactions. Principal concerns in the development of efficient BDS include the environmental impact of the carrier, mechanical and chemical robustness to avoid unintended leaching into crops and, control of the loading and release rate for efficient biocidal effects^2^.

Synthetic silica nanoparticles have been widely investigated as carriers for delivery systems, both for pharmaceutical^4^ and agrochemical applications^2^. These particles are particularly attractive for upcoming smart delivery, due to their high specific surface area^5^, engineerable surface chemistry^6^, biological inertness to both flora and fauna^7,8^ and thermal stability^6^. However, the bottom-up synthetic processes to obtain silica nanoparticles are complex and use non-renewable precursors through a process that generates highly hazardous biproducts and toxic intermediate reactants such as ferrosilicon (FeSi) and silicon tetrachloride (SiCl_4_). Moreover, FeSi is responsible for a reasonable fraction of air pollution and cause severe health hazards around production sites^9,10^, while SiCl_4_ is highly toxic^11^. We previously reported on biomass-derived silica particles (biogenic silica) that can be used as an affordable, ecofriendly and efficient alternative for BDS carriers^12^. Biogenic silica particles have a high surface area (up to 400 m^2^·g^−1^) and their surface chemistry is identical to the synthetic counterparts. Biogenic silica consists of a fractal-like network of 8–10 nm subunits hierarchically organized into agglomerates with sizes ranging from 100 nm to 4 µm, depending on the treatment applied for dispersion^13–15^. The facile surface modification of silica can be translated to that of biogenic silica structures as a mean to tune the dynamics of biocide release. Previously, this has been achieved in BDS, for instance, by gate keeping^16^ and near-infrared induced release^17^. However, these sophisticated approaches are costly and complicated for implementation, limiting their direct application as BDS. As Siepmann and Siepmann^18^ pointed out, the carrier/drug interface interaction plays a fundamental role in the drug release mechanism. Therefore, by promoting different carrier/biocide interface interactions through surface modifications, the release rates can be tuned. Furthermore, they can render BDS more responsive to external stimuli such as pH, temperature and ionic strength. Herein, through specific surface modifications of biogenic silica, we propose and develop a generic strategy to obtain “green” BDS with a tunable and responsive release. Such features are essential in order to minimize the amount of biocides delivered for efficient pest control as a function of soil or crop type as well as climatic conditions.

We use thymol as a model biocide as it is one of the most interesting and highly investigated biopesticide^1^. Thymol inhibits the proliferation of food fungi^19^, wood decay^20^, mold^21^, crop pests^22^, spoilage yeasts^23^, mosquitoes and beetles^24,25^. Additionally, as it is sourced from plants belonging to the *Lamiaceae* family, such as *Thymus* and *Origanum* species, its loading in biogenic silica would result in a fully biomass-derived BDS. We have developed an efficient, low energy loading strategy by using the appropriate solvent to reach dynamic loadings of up to 100 mg thymol per gram of biogenic silica. The release rate was controlled by using two additional types of biogenic silica, namely, those with high density of either amines or carboxylic acids. We demonstrate, via thermal decomposition patterns and surface analysis, the high stability of thymol loaded onto the biogenic silica. In fact, this was significantly improved in the case of the functionalized biogenic silica. We demonstrate that the thymol release rate is responsive to salinity, pH and temperature and highly dependent on the type of biogenic used as support. Lastly, we show the strong relationship between the release rate and the biocidal activity. A significantly more focused and pronounced biocidal activity is demonstrated for thymol-loaded biogenic silica when compared with unloaded systems.

## Results and Discussion

### Morphological features and evaluation of biocide loading efficiency and payload in biogenic silica

**Figure 1:**
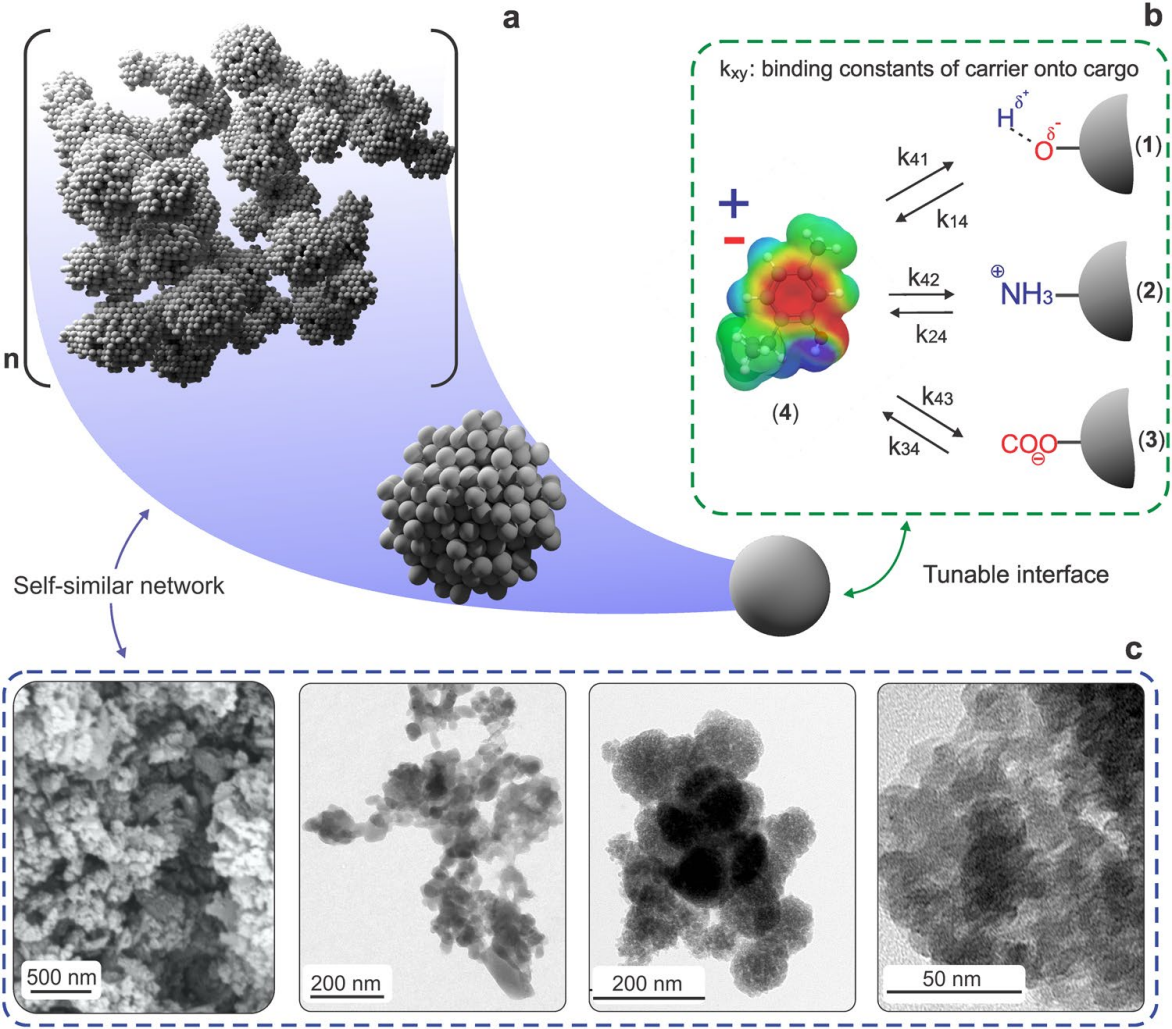
Schematics of the self-similar architecture of biogenic silica showing a hierarchical organization of particles with high surface area and accessibility (**a**). Various interactions between the carrier (biogenic silica) and the biocide (thymol) are expected to promote dynamic loading and release (**b**). Transmission electron microscopy and scanning electron microscopy images indicate the characteristic architectures of biogenic silica particles, drawn also in (**a**), covering a wide range of characteristic sizes, with particles of tens of nanometers (far right, TEM image) to organized, larger structures shown on the far left as an SEM image with a scale bar of 500 nm (**c**).

The deposits of silicon in the cell walls of horsetail plant are naturally nanostructured^26^, and they can be deconstructed into particles, the biogenic silica (Fig. 1c). We have shown that the top-down route for isolation of biogenic silica particles results in a polydisperse dispersion^27^. Such polydispersity of biogenic silica could be reduced by simple size fractionation, via sequential sedimentation. In this case, the biogenic silica can be separated into sedimenting fractions with particle sizes of *ca*. 10 µm, a significant fraction of particles <5 µm, and a colloidal fraction with particles with a predominant diameter of *ca*. 200 nm (Supplementary Fig. S2). The larger particles had a sedimentation rate of *ca*. 1 h, which indicated that they did not contain dense clusters of silica but a rather porous structure that was homogeneously distributed.

Across the range of particles composing the as-obtained dispersion, the BSiO_2_ particles formed nano-architectures hierarchically organized from 8–10 nm primary units that assembled into 30–40 nm spherical aggregates, further associated into clusters with sizes from 200 nm to more than 10 µm. The BSiO_2_ natural assemblies result in a self-similar architecture, Fig. 1a, experimentally confirmed in Fig. 1c. The specific surface areas (SSA) of unmodified, amino, and carboxyl-functionalized BSiO_2_ particles, obtained by the Brunauer–Emmett–Teller multipoint model, were 325 ± 2.5, 280 ± 7.5 and 269 ± 10.5 m^2^·g^−1^, respectively. Thus, the surface functionalization led to a marginal reduction in the SSA of BSiO_2_, which is in agreement with previous reports on synthetically-modified mesoporous silica^16,28,29^. Steric effects from larger moieties at silica surfaces are likely the main factor in the reduced SSA.

Amine and carboxyl groups on the surface of the biogenic silica were confirmed through XPS analyses (Supplementary Fig. S3). The electrostatic potential envelop of thymol at equilibrium conformation highlights a strongly polarized molecule with a shifted quadrupolar moment, leading to delocalization of strong positive and negative areas. Thus, besides van der Walls and H-bonding, dipole-dipole and electrostatic interactions can take place between thymol with the native or modified silica (Fig. 1b).

**Figure 2:**
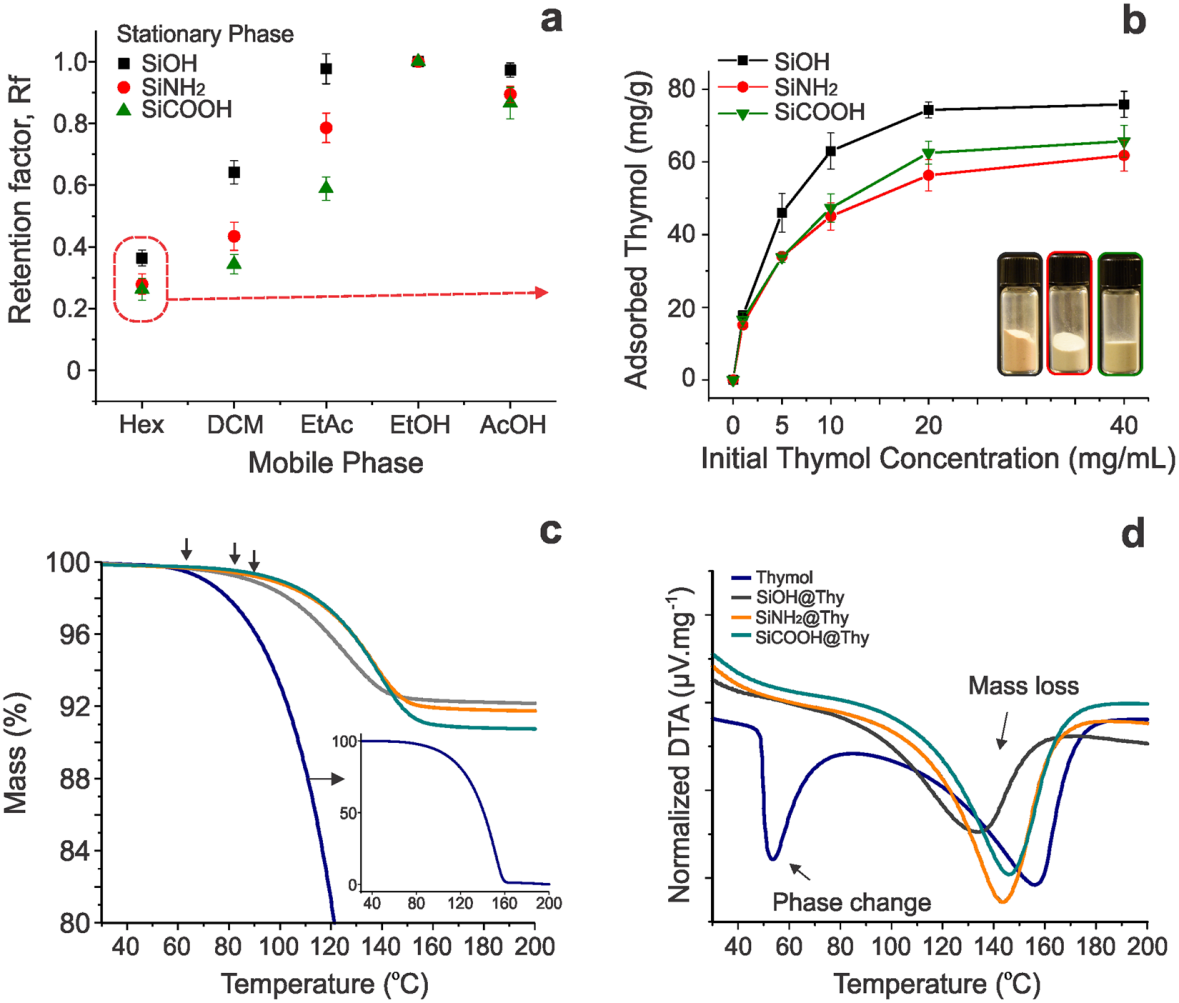
Retention factor (Rf) obtained from thin layer chromatography experiments using SiOH, SiNH_2_ and SiCOOH as stationary phases. The mobile phase included hexane (Hex), dichloromethane (DCM), ethyl acetate (EtAc), ethanol, (EtOH) or acetic acid (AcOH) (**a**). Isotherms for thymol adsorption at 25 °C on the biogenic silica carriers, SiOH, SiNH_2_ and SiCOOH (**b**). TGA curves showing the initial higher thermal stability of thymol adsorbed on BSiO_2_ (**c**), and DTA curves highlighting its loading in a molecular level (**d**).

We first evaluated the affinity of thymol with the various silica particles that were used as the stationary phase in thin layer chromatography (TLC). As a result of the poor water solubility of thymol, distilled water was not suitable to elute the oil (Rf = 0), regardless the type of silica stationary phase used. This emphasizes the need for loading thymol in silica using an appropriate solvent, so that the cohesive crystal lattice energy that limits dissolution can be overcome and, thus, to obtain favorable release^30^. Regarding organic solvents, the amino- and especially carboxyl- modified biogenic silica provided strong interactions with thymol, as shown by the respective, comparatively lower thymol retention factors (Fig. 2a). In a typical TLC experiment, the eluting process is based on the competition between solvent and analyte for binding sites on the stationary phase, which occurs in terms of polarity similarity^31^. A parallel can be directly drawn between TLC experiments and a system consisting of biogenic silica particles dispersed in thymol solution. The polarity of the solvent was fundamental for loading (Fig. 2b) or quantifying thymol on the different carriers. The strong affinity of thymol to polar solvents, in this case ethanol, allowed to effectively quantify its payload in the BDS. Indeed, a significant amount of thymol could be freed from the silica in ethanol. On the other hand, the non-polar n-hexane was able to dissolve thymol, but promoted spontaneous thymol loading on silica (Fig. 2b). The dynamic biocide loading capacity (maximum releasable payload) of the BSiO_2_ particles varied from 70 to 50 mg·g^−1^, a similar range was reported for synthetic mesoporous silica nanoparticles with much higher surface area (up to 1000 m^2^·g^−1^)^16,32,33^. Thus, the remarkably high loading capacity of biogenic silica is derived from its high-percolating fractal architecture. This is in contrast to other systems, for example, electrospun fibers displaying high surface areas but with limited loading, e.g., 2.5%. for blends of poly(ɛ-caprolactone) and poly(lactic acid)^34^.

Compared to the thymol loading in the unmodified BSiO_2_ (SiOH@Thy), the dynamic biocide payload (extractable) was statistically lower for the functionalized biogenic silica particles (SiNH_2_@Thy and SiCOOH@Thy, Fig. 2b, Table S1); however, the total payload was higher in these latter cases. The peak of thermal degradation of free thymol occurred at 160 °C (Fig. 2c,d) and thermal degradation of the functional groups on the biogenic silica (modified and unmodified) occurred above 200 °C (Supplementary Fig. S4). Therefore, the total thymol loading could be estimated by heating 100 mg of all three types of dried BDS at 200 °C for 24 h. All the mass loss in this procedure was attributed to thymol. By this method, the total amount of biocide loaded in the BDS was estimated to be 82.5, 99.5 and 119.0 mg·g^−1^ for SiOH@Thy, SiNH_2_@Thy and SiCOOH@Thy, respectively. The extractable and the actual amount of thymol loaded in the unmodified carrier were similar (75 and 82.5 mg·g^−1^, respectively, 9.3% of non-released thymol in EtOH); however, for the functionalized particles, the difference between the loaded and the releasable biocide increased by 63 and 83% for the amino- and carboxyl-modified BSiO_2_ particles, respectively. The lower Rf of thymol on the SiCOOH carrier is indicative of strong interactions between the components.

Interestingly, the temperature of initial mass loss of the thermograms significantly increased when comparing free thymol with the BDSs. Compared to free thymol, the temperature for the initial mass loss of the SiOH@Thy system shifted from 57 to 72 °C (Fig. 2c). This temperature was even higher, *ca*. 95 °C, for the SiNH_2_@Thy and SiCOOH@Thy systems. However, thymol absorbed on biogenic silica displayed a DTA peak corresponding to mass loss that shifted toward lower temperatures; meanwhile, the peak corresponding to phase change could not be observed (Fig. 2d). This indicates that thymol adsorption on all the carriers occurred with a strength that is beyond physical interactions. The absence of phase change was also observed, for instance, when thymol was complexed in β-cyclodextrin^35^. Specifically, for free thymol, latent heat is required firstly to produce phase change from crystal to liquid (oil), then to break its cohesive energy and to promote volatilization/degradation. We speculate that in a BDS, the thermal energy is only consumed to volatize/decompose the adsorbed thymol, which results in the shift toward lower temperatures. Moreover, the observed temperature shift was smaller in the case of the modified particles, indicating that a higher energy is required to overcome the silica-thymol interactions prior to degradation/volatilization. More specifically, the mass loss prior to volatilization of thymol adsorbed on silica (prior to the mass loss peak) occurred at lower temperature for unmodified silica, followed by higher temperatures in the case of amino- and carboxylic acid-functionalized silica. This further highlights that the strongest affinity of thymol is for carboxylic acids, followed by those with amine and with hydroxyl-surfaces.

### BSiO_2_-thymol based BDS

The FT-IR spectrum of the unmodified biogenic silica particles presented characteristic peaks at 1650 and 800 cm^−1^, which are related to different vibration modes of the hydroxyl bonds in silica. The peaks at 1100 and 460 cm^−1^ correspond to the asymmetric stretching and bending vibration of Si-O-Si group, respectively^36^. The region at *ca*. 2935 cm^−1^ (Supplementary Fig. S5) refers to the multiple arrangements of C-H stretching vibration in amine and carboxyl-functionalized silicas. Low intensity peaks appeared at 1705 and 1565 cm^−1^ for the SiCOOH carrier corresponding to the stretching vibration of the C=O from the carboxylic acid and amide groups, respectively. The mid-IR fingerprint for thymol comprises the wavenumber range between 900 and 1400 cm^−1^. In this region, characteristic, key peaks for thymol appear at 945, 1087 and 1290 cm^−1 ^^37^. In addition, characteristic peaks for aromatic rings corresponding to C-C=C symmetric and asymmetric stretch vibration at 1585 and 1460 cm^−1^, respectively, were also useful to identify thymol in the BDS^38^.

**Figure 3:**
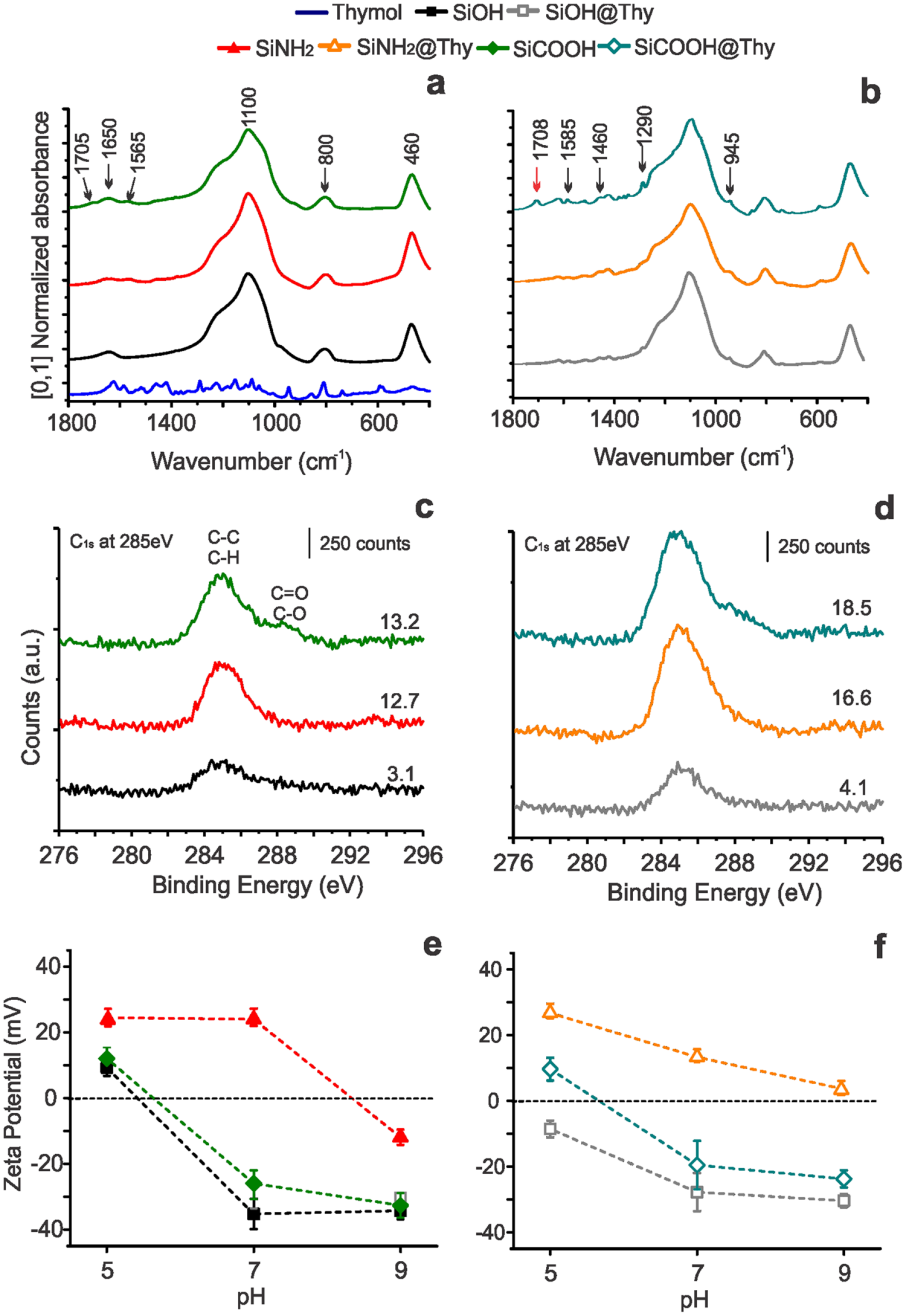
FTIR spectra of the biogenic silica carriers (**a**) and prepared BDS (**b**). C_1s_ high-resolution XPS spectra of the unmodified and modified particles (**c**) and the BSiO_2_-thymol based BDS (**d**) with detail of the apparent carbon composition at the BDS and carriers surface. Zeta potential measurements to verify the charge of the silica surface before (**e**) and after thymol loading (**f**) as a function of pH.

The peaks related to thymol chemical structures are more defined in the SiCOOH@Thy spectrum. This result corroborates with data from thermal analyses (Fig. 2c,d) and the apparent surface chemical composition obtained via XPS (Fig. 3d). These peaks are also observed for SiOH@Thy and SiNH_2_@Thy systems, but with a lower intensity (Fig. 3b). The spectrum of the SiCOOH@Thy sample also showed an increase in intensity of the peak at 1708 cm^−1^, which is related to C=O stretching. Also, this peak was observed at a slightly more delocalized wavenumber than it observed from the carboxyl-based carrier. Hypothetically, ester bonds could have occurred between carboxylic acid termination of the silica surface and hydroxyl groups from thymol^39,40^. The formation of this bond partially explains the higher stability of thymol on the SiCOOH carrier; however, it is important to emphasize that the amide groups present in SiCOOH could form strong H-bonds with thymol, which could also contribute to an enhanced thymol stability.

The proposed carbon-oxygen interactions between SiCOOH and thymol also appeared in the C_1s_ high-resolution XPS spectra of the SiCOOH@Thy (Fig. 3c,d, Supplementary Fig. S6). The C_1s_ XPS spectrum had a peak at 285 eV, corresponding to C-C and C-H moieties^41^. Contributions at higher binding energies (increase by *ca*. 3.5 eV) usually appears as shoulders and are associated to carbon-oxygen interactions^41^. The carboxyl groups originally presented on the modified silica carrier contributed to the C_1s_ spectra as an important component appearing as a shoulder at 288.5 eV. The C=O contribution in the carboxyl-based BDS appeared at different binding energy (287.5 eV), which indicate that the interaction between COOH termination and thymol can result in the formation of different carbon-oxygen interactions.

The apparent carbon composition at the surface of the BDS, measured through XPS, corroborated the higher stability of thymol on BDS prepared with the functionalized particles (Fig. 3c,d). Nevertheless, thymol, as a phenolic monoterpene, is volatile^42^ and therefore the XPS quantification of thymol on the surface is compromised because the high vacuum needed for analysis. This can be expected to accelerate thymol volatilization, unless it strongly interacts with the surface. For instance, the disparity between the thymol content quantified in the SiOH carrier after its extraction (Fig. 2b) and XPS confirms this hypothesis. Thus, despite the fact that SiOH@Thy has a high thymol payload, in this system the biocide is less stable than in the functionalized particles.

All carriers showed negative zeta potential at pH 9, while at acid and neutral pH the zeta potential varied according to the surface chemistry of the particles (Fig. 3e). Both SiOH and SiCOOH carriers presented an isoelectric point (IEP) between pH 5 and 6, while the SiNH_2_ carrier had an IEP between pH 8 to 9. IEP and zeta potential as a function of pH results from the contributing charged groups on the silica surface. The differences observed for the IEP and zeta potential are attributed to the specific mechanisms of (de)protonation of the silanol, carboxyl and amino groups. Protonation of the amines seems to occur between pH 8 and 9 whereas deprotonation of the COOH groups would occur between pH 5 and 7.

After thymol loading, the zeta potential of the resultant BDS clearly shifted towards zero for all particulate systems. It means that both positive and negative charges at the silica surface were partially shielded by adsorption of the uncharged thymol. As we showed, thymol presents both positive and negative electrostatic potential and is highly polar in nature (Fig. 1a). The zeta potential of the cargo and its carrier as well as the resultant BDS has significant implications to the responsiveness of the delivery, especially over pH changes^43^.

### Biocide release profiles, kinetics and thermodynamic considerations

**Figure 4:**
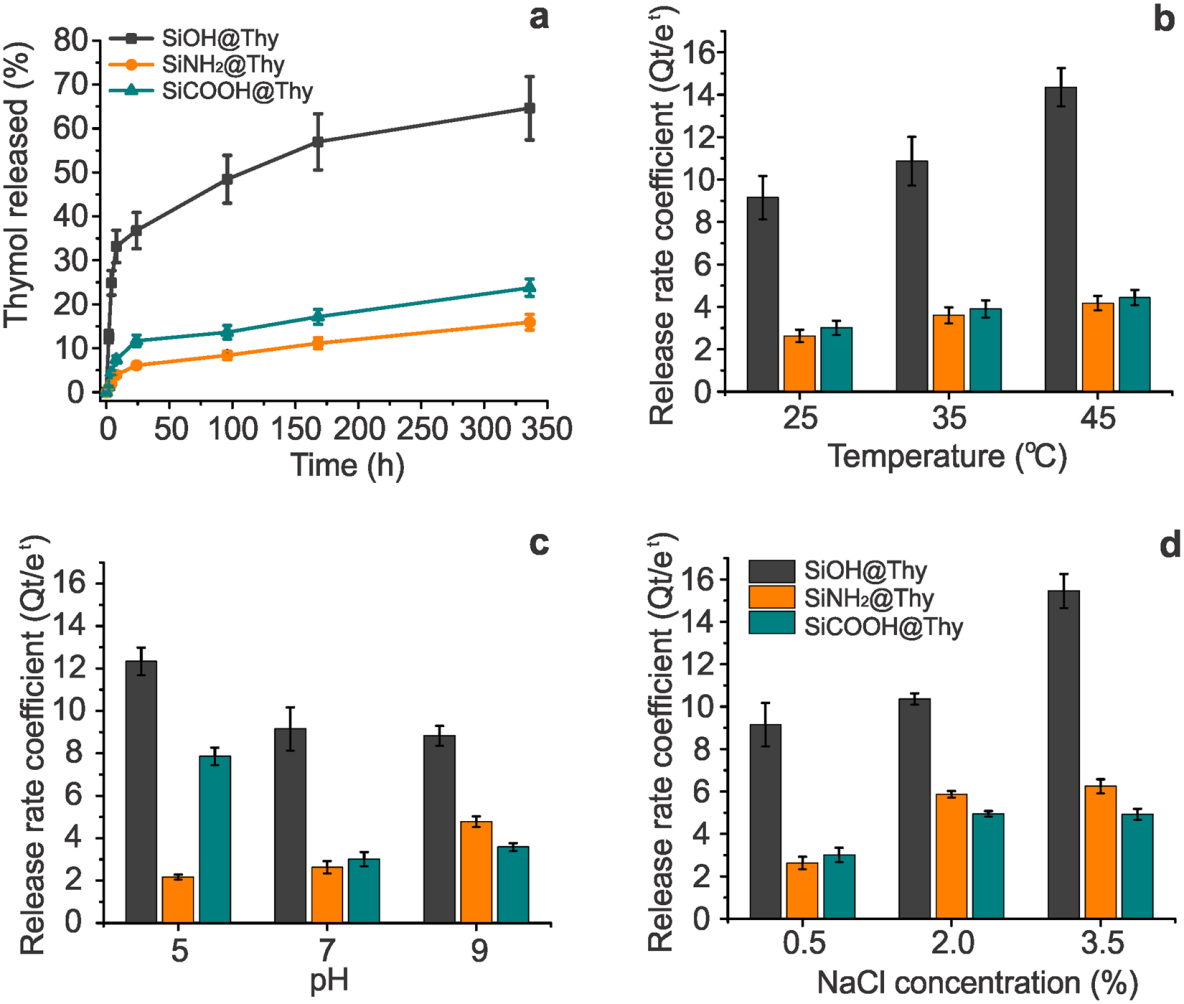
Thymol release profiles under regular aqueous media conditions (25 °C, pH 7, NaCl 0.5%) showing release rates according to different interactions (**a**). Release rate coefficients, *k*, obtained after linearization of the experimental release profiles using a modified Elovich equation in order to investigate effects of temperature (**b**), pH (**c**) and salinity (**d**) on the thymol release.

The prepared BDS were designed to control thymol dissolution in water by tailoring the carrier-biocide interactions. The release profiles behave similarly, regardless the size fractions of the carrier particles (from very large sedimenting particles to colloidally stable particles, Supplementary Fig. S7). This highlights that the self-similar nanostructure of the particles is a key feature of this biogenic silica-based BDS. In all release profiles, thymol releases from the carrier through two different stages: initial burst stage, observed in the first 24 h, followed by a slow-release stage, from 24 to 350 h (Fig. 4a). Equilibrium was not achieved for none of the BDS, at least during the observation time of 350 h, suggesting that sustained release may occur for even longer times. When loading thymol in zein films, Nobile *et al*.^44^ found that equilibrium was reached after 20 h, even though different thymol payloads were considered. In addition, the release profiles obtained for polymeric blends presented similar stages as those discussed here but at higher rates^34^. SiNH_2_@Thy and SiCOOH@Thy systems released statistically lower amounts of thymol (Supplementary Table S2), which is a result of their stronger carrier-biocide interactions (Figs 2 and 3). The initial burst release is attributed to the thymol sequential layers over its first layer on the silica surface. Considering thymol molecular size as well as the results observed from XPS analyses (Fig. 3c,d), the adsorbed amount of thymol was likely to result in multiple layers of thymol being adsorbed.

Several kinetic models often applied to describe cargo delivery^12,16,17,45,46^ were assessed to fit experimental data from thymol release experiments (Supplementary Table S3). We considered the initial concentration of thymol in the release media to be zero, and that only desorption occurred, thus simplifying the integrated form of the equations. The Elovich kinetic model fitted the thymol release profile very well (coefficient of determination over 0.96, Supplementary Table S3). Elovich’s fit highlights that thymol delivery follows a logarithmic, time-dependent, decay. This means that the differential amount delivered over time (determined by *k*) decreased proportionally to the amount released in previous stages. This mechanism is a result of the fractal network of the carrier that allows fast solvent (water) diffusion into and out of the carrier. In this condition, the thymol in the more exposed (outer) regions of the carrier is released promptly, while the thymol located in the inner regions of the carrier is released with more difficulty. We speculate that over the release time, the amount of thymol along the carrier cross-section became more homogeneous; thus, the release rate decreased due to a slower diffusion.

The release rate coefficient, *k*, was determined from the slope of the Elovich linearized plot (Supplementary Fig. S8b). *k* was used to investigate the responsiveness of the BDS over pH, temperature and salinity (Fig. 4b–d). Overall, at regular conditions (25 °C, pH 7, NaCl 0.5%), the release rate was three-fold higher in the SiOH@Thy system than in the BDS prepared with functionalized particles. By using carrier mixtures, the delivery rate can be change and allow even finer tuning of the release dynamics. This is illustrated in Supplementary Fig. S8a for the theoretical release rate obtained from mixtures of SiOH@Thy and SiNH_2_@Thy.

The release rate of thymol from all prepared BDS increased as the temperature increased (Fig. 4b). The temperature factor was responsible for statistically significant changes in *k*, in which changes are dependent on the surface (-OH, -NH_2_, and -COOH) (Supplementary Table S4). The effect of temperature, in terms of percentage variation, was higher for SiNH_2_@Thy, followed by SiCOOH@Thy and SiOH@Thy. The *k*_45 °C_/*k*_15 °C_ ratio was 2.47, 1.86 and 1.55, respectively. An additional release profile at 15 °C was carried out in order to precisely obtain the activation energy, which was calculated to be 350, 235 and 125 J·mol^−1^ (Supplementary Fig. S8c).

The pH plays an important role in the release rate of thymol (Fig. 4c). We demonstrated that *k* is statistically affected by pH changes, having significant effects between pH for a given surface (-OH, -NH_2_, and -COOH) or between surfaces considering a fixed pH (Supplementary Table S5). When considering principally electrostatic interactions, the variations in the release rate observed at various values of pH can be understood considering that thymol is a phenolic molecule with an acid dissociation constant pKa of *ca*. 9. The release rate of the SiOH@Thy system became slower in a pH range where the particles presented a net negative charge (Fig. 3f), suggesting that the Si-OH surface, below pH 7, leads to faster release when compared with Si-O^−^. In the BDS prepared with the amine-functionalized particles, the protonation of the NH_2_ to NH_3_^+^, below pH 9, was essential to understand the behavior of *k* over pH changes. At pH 5 the NH_3_^+^ (carrier) and OH (biocide) did not interact, leading to the fastest release. Meanwhile, the NH_3_^+^ and O^−^ (pH 7) contributed to the slowest release because of the several interactions that can occur in these conditions (Fig. 1b). At pH 9 a weak interaction driven by NH_2_ and O^−^ from the carrier (negative zeta-potential) occurred, resulting again in a faster release. Regarding the SiCOOH@Thy system, the release rate at pH 7 and 9 were similar, due to negative charge at the carrier surface and biocide. Looking only at electrostatic interactions, it was not expected much higher release rate at pH 5, since COOH and OH interaction is as weak as COO^−^ and O^−^. However, acid media could act as a catalyst for hydrolysis of ester bonds, which hypothetically could have occurred between thymol and the SiCOOH carrier^39,40^. Considering this to be true, we propose that the highest release rate of thymol from the SiCOOH@Thy system would be at pH 5, which was the experimental observation. In the discussion of the effect of pH on release rate we principally indicated the stronger, electrostatic interactions but other interactions such as van der Walls and H-bonding may play important role as well.

Salinity, like temperature and pH, promoted statistically significant changes in *k* (Supplementary Table S6). When the salinity is systematically increased, from 0.5 to 3.5% of NaCl, the release rate coefficient for SiNH_2_@Thy increased by up to 2.5 times and up to *ca*. 1.6 times for the SiOH@Thy and SiCOOH@Thy systems. The strong effect of the ionic strength on the thymol release from the amine-based BDS, indicates that at the carrier-biocide interaction was strongly driven by electrostatic interactions. On the other hand, the less marked effect of ionic strength on the release rate in the SiOH@Thy and SiCOOH@Thy systems suggests that the carrier-biocide interactions were not only driven by electrostatic effects but also by van der Walls, short-range dipole-dipole (*e.g*. through quadrupole) and multiples H-bonding interactions.

### Biological activity of biogenic silica BDS

**Figure 5:**
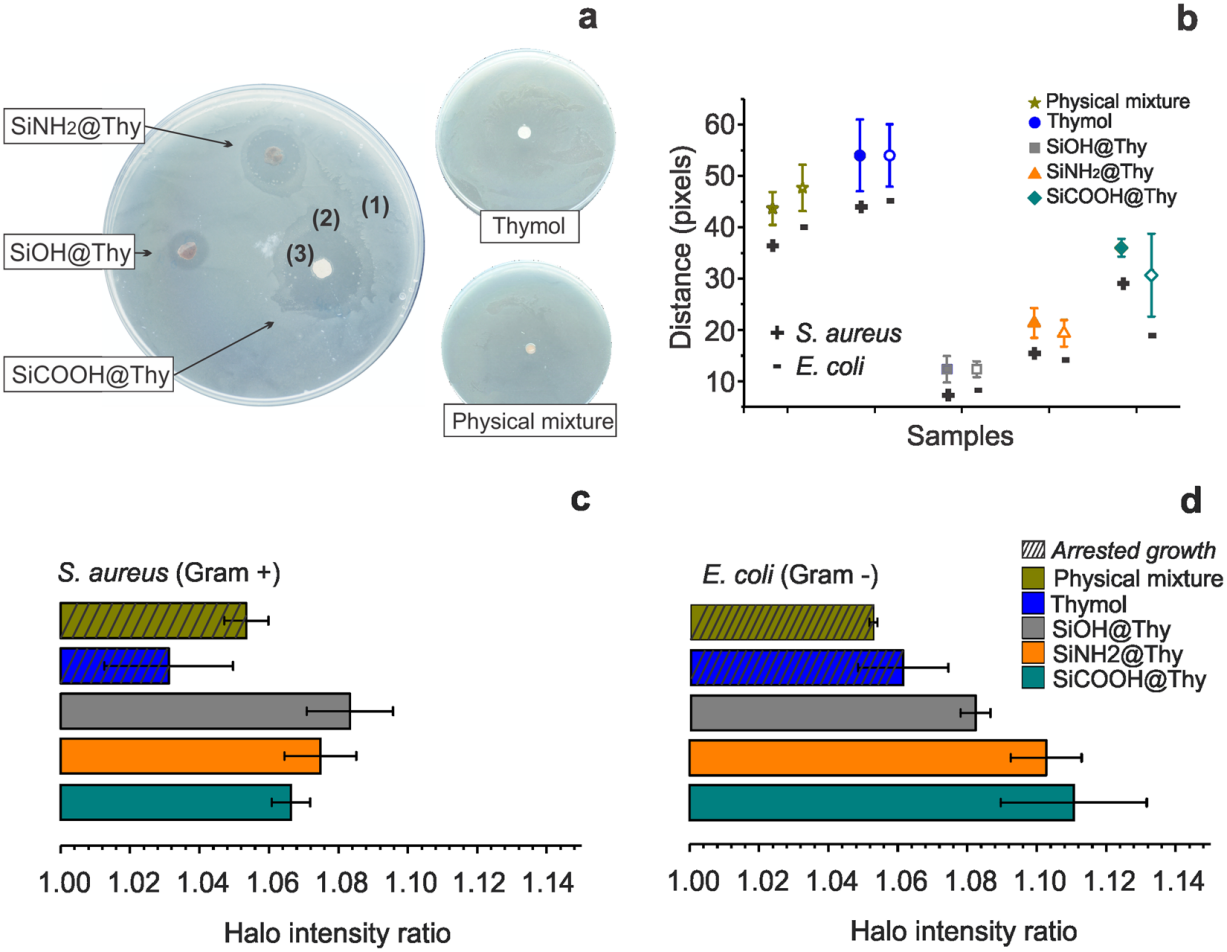
Results of typical agar diffusion tests using *Staphylococcus aureus* plates treated with SiOH@Thy, SiNH_2_@Thy and SiCOOH@Thy BDS discs and control samples, i.e., thymol oil adsorbed on paper and thymol oil blended with silica in the dry state (physical mixture) (**a**). Length of the bacteria-free zone measured in pixels for both *S.aureus (gram*+*) and E.coli* (gram −) (**b**). Ratio between halo intensity of the zone colonized by bacteria and bacteria-free zone of the *S.aureus* (**c**) and *E.coli* (**d**) plates.

The biocidal activity of the prepared BDS was assessed against *Escherichia coli* (gram negative) and *Staphylococcus aureus* (gram positive). The activity of free thymol against these organisms was previously reported^25^ and confirmed in this study (Fig. 5). The agar diffusion tests allowed us to qualitatively assess the bioactivity of the obtained BDS (Fig. 5a) and to highlight a higher activity than that of neat thymol.

Three well-defined zones of bacterial growth were observed in the agar diffusion plates for *S. aureus* (Fig. 5a) and *E.coli* (Supplementary Fig. S9). In order to provide more quantitative insights, we measured the distance of the halo and calculated the ratio between the halo intensity of all three zones. The first zone corresponded to areas where the bacteria growth was not inhibited by the biocide, Fig. 5a(1). An intermediate region was identified for the SiNH_2_@Thy and SiCOOH@Thy pellets for both *S. aureus* and *E.coli* plates. The contrast in this zone was not strong, which corresponded to partially inhibited growth of bacteria Fig. 5a(2).The third region corresponded to complete inhibition of bacterial growth, which could be easily identified for BDS specimens as no bacterial growth could be observed in this region, Fig. 5a(3). In contrast, free thymol oil or thymol physically mixed with silica presented a greater diameter of partial inhibition. Importantly, neither free thymol nor thymol mixed with silica completely inhibited bacterial growth. The same contrast in the partially inhibited zone (2) of BDSs were observed for both controls. The average halo intensity ratio for the control samples showed values ranging from 1.01 to 1.06 for the *S. aureus* plates and 1.05 to 1.075 for *E.coli* plates. As a comparison, the same ratio for the various BDS achieved values ranging from 1.07 to 1.09 and 1.08 to1.11 for *S. aureus* and *E.coli* plates, respectively. Compared to its application in nanostructured BDS, thymol was statistically less active in its free form. The physical mixture of thymol and unmodified silica further corroborated the improvement of thymol bioactivity through molecular adsorption on the nanostructured silica carrier. By doing so, the availability of the biocide was enhanced, thus improving a sustained biocidal activity, which is a factor that deserves further studies. On the contrary, non-dissolved thymol encapsulated in cellulose-based matrices, via emulsion routes, had the same minimum inhibitory concentration values when compared to its free form^47^.

Among the BDS tested, a statistically higher activity (higher ratio and distance) was noted (Supplementary Table S7), as expected for those with higher thymol loading, SiCOOH@Thy and SiNH_2_@Thy. From this result, we noticed that the biological activity of thymol may be also attributed to the molecules that were observed not to be delivered in the release conditions reported in Fig. 4, i.e., the fraction of thymol strongly tethered on the carrier surface. Thus, even the BDS with a low biocide release performed best at controlling bacterial growth.

## Conclusions

We propose biogenic silica carriers to support large thymol loadings that take advantage of a complex (fractal) structure, which afforded controlled and tailorable release. The biocide payload in the biogenic silica is comparable with mesoporous silica (*ca*. 100 mg per g of carrier), even considering specific surface areas twice higher for the last. The high accessibility of the surface area in such self-similar network was key to this achievement. Here, biocide delivery systems (BDS) that used thymol as model compound were shown as suitable and inexpensive platforms to obtain biocide dynamic loading and release. This was accomplished by tuning the surface charges on the biogenic silica upon functionalization with amine and carboxyl groups. Amine and carboxyl groups promoted stronger interactions between carrier and biocide, resulting in controllable and responsive release as well as higher thermal stability and localized antibacterial activity. Such systems are relevant for large-scale applications and show promise beyond the performance measured for neat or free biocide forms.

## Materials and Methods

Most of the data generated or analyzed during this study are included in this published article (and its Supplementary Information files). The rest of the raw data generated during and/or analyzed during the current study are available upon request from the corresponding authors.

### Materials and Chemicals

Thymol (Thy), Maleic anhydride, 3-Aminopropyl-triethoxysilane (APTES), sodium chloride (NaCl), sodium hydroxide (NaOH), hydrochloric acid (HCl), calcium sulfate hemidrate, lysogeny, tryptone, yeast extract, agar, starch, casein, beef extracts and organic solvents were purchased from Sigma-Aldrich. Biogenic silica particles (BSiO_2_) were isolated from *Equisetum arvense* (horsetail) as previously described. Briefly, the process involved hydroalcoholic pretreatment (1:1 H_2_O:EtOH) followed by acid hydrolysis (H_2_SO_4_ 2% w·v^−1^, 100 °C, and 1 h) and calcination (650 °C for 1 h)^14^.

### Design and synthesis of the BSiO_2_-thymol BDS

The BSiO_2_ was amine-functionalized by using APTES^48^: 1 g SiOH and APTES (4 mL) were placed in a flask containing 100 mL toluene. After ultrasonication (30 min), the dispersion was refluxed at *ca*. 110 °C for 6 h, and then subjected to centrifugation (5000 rpm, 10 min), sequential washing with toluene (3x) and absolute ethanol (2x), and drying at 103 °C. The obtained particles are thereafter referred to as “SiNH_2_”. Carboxyl-functionalized surface was achieved by subsequent amidation reaction of SiNH_2_ particles using maleic anhydride^49^: 1 g SiNH_2_ and maleic anhydride-DMF solution (40 mL at 2 mol·L^−1^) were placed in a flask, ultrasonicated (30 min), and kept under stirring. After equilibrating for 24 h, the suspension was centrifuged (5000 rpm, 10 min), sequential washed with DMF (3x) and absolute ethanol (2x), and dried at 103 °C. The obtained particles are thereafter referred to as “SiCOOH”.

The biocide-carrier-solvent affinities were investigated by using the thin layer chromatography (TLC) technique. The TLC plates preparation and experiments followed well-known procedures^50^. Unmodified and surface-functionalized BSiO_2_ particles were used as the stationary phase. Organic solvents with different polarity were used as the mobile phase. The carrier-biocide interactions were compared by calculating the retention factor (Rf) of thymol on silica over solvent changes.

First, 200 mg of BSiO_2_ was suspended in 10 mL n-hexane, submitted to indirect ultra-sonication for 30 min, and placed in a reciprocal shaker at 100 rpm and 25 °C. Then, under continuous mixing, 10 mL of thymol solution was added dropwise; the flask was sealed and left to equilibrate for 24 h. The thymol solutions were prepared in n-hexane at 1, 5, 10, 20 and 40 g·L^−1^ to optimize the loading procedure. The BSiO_2_-thymol based BDS were recovered after centrifuging the suspension at 2000 rpm for 1 min and further drying it at 80 °C for 4 h.

### Analytical and morphological characterization of the BSiO_2_-thymol BDS

The biocide payload in BDS was assessed through an UV-adsorption calibration curve at 275 nm. The surface chemical features of the BDS were elucidated by X-ray photoelectron spectroscopy (XPS) using a VG Microtech ESCA 3000 spectrometer. The equipment operated at 3 × 10^−10^ mbar, with Mg Kα radiation as excitation source (photo energy at 1 253.6 eV). The C_1s_ binding energy was set at 285 eV to use as internal reference for compensating for surface charging effects. The functional chemical groups and chemical interactions of the BDS were investigated by using Fourier transform infrared spectroscopy (FTIR). FTIR spectra were acquired on a Bruker Tensor 37 FTIR unit, using KBr pellets, at a nominal resolution of 4 cm^−1^ (64 scans in the 4000 to 400 cm^−1^ wavenumber range were accumulated and averaged). Thermogravimetric (TGA) and differential thermal analysis (DTA) were applied to investigate the effect of the interface interactions on the thermal behavior of thymol. TGA and DTA experiments were carried out in a DTG-60 Shimadzu equipment using N_2_ atmosphere with gas flow of 50 mL·min^−1^, temperature range from 25 to 200 °C, and a heating rate of 10 °C·min^−1^. Zeta potential of the starting BSiO_2_ particles and the resultant BDS were examined over pH changes by using Zetasizer ZS NanoS90 (Malvern). The reported average results of the zeta potential were determined from at least five measurements. The pH was adjusted by the addition of 0.01 mol·L^−1^ HCl or NaOH solutions. The representative morphological features of typical BSiO_2_ particles were studied by using a transmission electron microscope (TEM) JEOL, model JEM-1200 EXII, and a field-emission gun scanning electron microscope (FEG-SEM) FEI Quanta 450. Also, an optical microscope Leica ICC50 HD, operating in bright field, was used to observe the morphological features of the settling, larger, fraction of the silica particles.

### Biocide release profiles and kinetics

The biocide release profiles were investigated under various conditions in the release media. Specifically, three pH values (5, 7 and 9), electrolyte concentrations (NaCl, 0.5, 2.0 and 3.5% w·v^−1^) and temperatures (25, 35 and 45 °C) were evaluated. The pH of the suspension was adjusted using the same procedure used for Zeta potential measurements. In a typical release experiment, filter paper envelopes (72 samples: 3 surfaces x 3 replicates x 8 time points) containing *ca*. 30 mg of BDS were placed in 1 L of dH_2_O at given pH, salinity and temperature. This methodology was applied to precisely ensure the same release conditions for sample comparison. Then, the envelopes were removed at fixed time intervals, from 0 to 350 h, and the amount of the residual thymol in the BDS was extracted with ethanol and quantified via UV calibration curve. The release profile data were acquired using triplicates. The Elovich kinetic model^51^ led to the best fitting and allowed evaluation of the release rate as a function of a single kinetic parameter (*k*). The activation energy (AE) of the biocide delivery was calculated with the Arrhenius equation.

### Biological activity of the BSiO_2_ particles supporting thymol

Suspensions at 20% solid content of each BDS, namely, SiOH@Thy, SiNH_2_@Thy, and SiCOOH@Thy, were prepared for readily casting of 50 µL volume on a cleaned polystyrene plate. The resulting pellet was 10 mg in mass and *ca*. 5 mm in diameter. For the control samples, we considered an average thymol payload in the BDS of 100 mg·g^−1^. Thus, 1 µL of undissolved thymol oil was casted on a 5 mm filter paper disc. In addition, we prepared a physical mixture of undissolved thymol and SiOH particles. In this case, first the SiOH pellet was prepared as mentioned above and then 1 µL of thymol oil was casted on it.

The antimicrobial effect of the BDS systems were tested using conventional agar diffusion experiments according to standardized Kirby-Bauer procedure^52^, against gram-negative (*Escherichia coli*) and gram-positive (*Staphylococcus aureus*) bacteria. Both strains were first pre-cultured on lysogeny broth (LB) agar plate at 37 °C for 24 h containing 1% w·v^−1^ tryptone, 0.5% w·v^−1^ yeast extract, 1% w·v^−1^ NaCl and 1.5% w·v^−1^ agar at pH 7.4. Cells were then cultivated in LB medium by inoculating one colony from pre-cultured LB-agar plates at 37 °C for 14 h until the optical density at 600 nm reached 0.5 (*ca*. 150 million cell·mL^−1^). For the diffusion tests the preparation consisted of 4 mm-deep Mueller-Hinton agar plates in a 100 mm petri dish containing 1.7% agar, 0.15% w·v^−1^ starch, 1.75% w·v^−1^ casein hydrolysate and 0.2% w·v^−1^ beef extract at pH 7.4. To inoculate the cells on the Mueller-Hinton plates, sterile L-shaped polypropylene cell spreaders were dip into the inoculum container and excess liquid was allowed to drain. Cells were then inoculated on the surface of Muller-Hinton plates by streaking the cell spreader evenly over the entire surface of the plate. The lid of the Petri dish was left open for 5 min to allow the surface of the agar plate to dry before placing the BDS discs. Plates were cultured for 24 h at 35 °C before examination. Halo intensity ratios and distance between unaffected growth and samples were calculated and used for discussion (Supplementary Fig. S1).

### Data availability

Data used in this research is available upon request.

## Electronic supplementary material

Supporting Information
