# Supplementary material for: Controlled biocide release from hierarchically-structured biogenic silica: surface chemistry to tune release rate and responsiveness
Source: Sci Rep. 2018 Apr 3;8:5555. doi: 10.1038/s41598-018-23921-2 (PMC5883024; doi:10.1038/s41598-018-23921-2)
Supplement: Supplementary file 1 — Supporting Information [file 41598_2018_23921_MOESM1_ESM.pdf]

Supporting Information

Controlled biocide release from hierarchically-structured biogenic silica: surface chemistry to tune release rate and responsiveness

Bruno D. Mattos[1,2,], Blaise L. Tardy[1], Mohammadi Pezhman[1], Tero Kämäräinen[1], Markus Linder[1], Wido H. Schreiner [3], Washington L. E. Magalhães[2,4], Orlando J. Rojas[1,5,*]*

[1]Department of Bioproducts and Biosystems, School of Chemical Engineering, Aalto University, FI-00076 Aalto, Finland.

[2] Integrated Program in Engineering & Materials Science, Federal University of Paraná, Polytechnic Center, Curitiba 81531-990, Brazil.

[3]Laboratório de Superfícies e Interfaces, Universidade Federal do Paraná, Curitiba 81531-980, Brazil.

[4] Embrapa Florestas, Estrada da Ribeira, Km 111, Colombo 83411-000, Brazil.

[5] Department of Applied Physics, School of Science, Aalto University, FI-00076 Aalto, Finland.

This Supporting Information document contains ten (10) figures and seven (7) tables in 20 pages (S1-S20).

Detailed information on the thin layer chromatograph (TLC) experiments. The thin layer chromatography (TLC) plates were prepared using the unmodified and surface modified biogenic silica nanoparticles as stationary phase. For this, 1.5 g silica was suspended into 30 ml of destilled water and ultrasonicated for 15 minutes, then 225 mg calcium sulfate hemidrate (15 wt% related to mass of silica) were added to the suspension and quickly homogenized. The suspension was dispensed on cleaned glass slides, which were allowed to air-dry overnight. Before use it, the plates were activated at 110°C for 30 minutes. Hexane (HEX), dichloromethane (DCM), ethyl acetate (EtAc), ethyl alcohol (EtOH), acetic acid (AcOH) and deionized water (dH_2O) were used as solvents to calculate the retention factor (Rf) of the thymol on silica.

Image processing for quantitative evaluation of the agar diffusion tests. The software ImageJ was used to create grayscale profiles of neighboring area of the discs observed in the biocidal assay (**Fig. S1**). To avoid external influence from the light reflection of the images, we have presented the results as the ratio between the intensity of region where bacteria colonies were unaffected by the BDS and the region next to the BDS pellets. Also, we measured the diameter of the pellets in pixels as an internal comparison for bactericidal assessment of the BDS. Three-line measurements were made spaced by 120°. The referred gray value was taken as an average of the entire zone in reference.

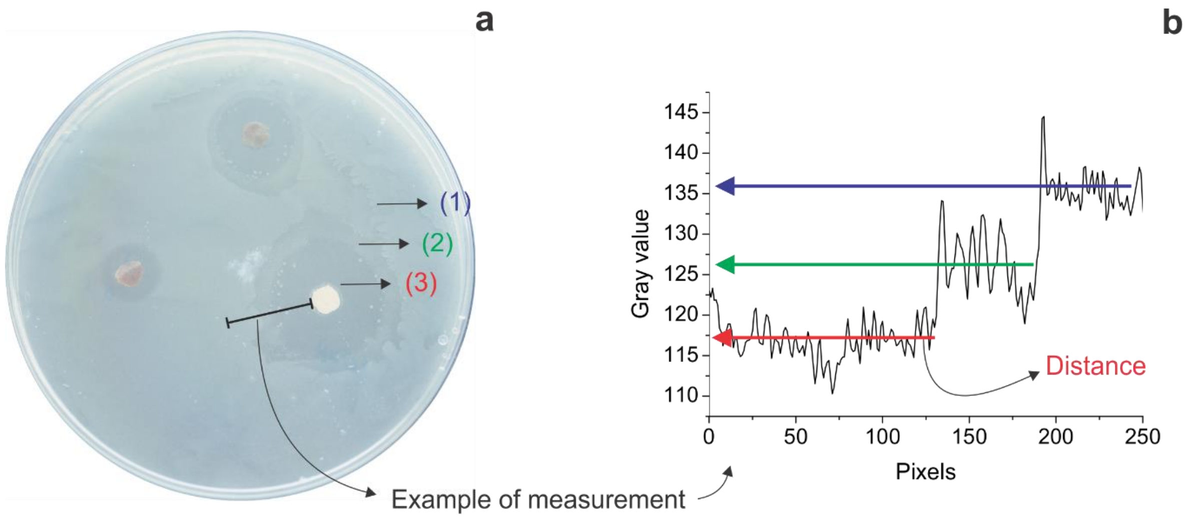

Fig. S1. Experimental procedure to calculate the quantitative bioactivity of the prepared BDS. Detailed information on the zones of interest (a) and halo intensity profiles (b), in order to exemplify the systematic procedure. 1: No inhibition zone; 2: partially inhibition zone; 3: complete inhibition zone.

Morphological features of biogenic silica and size fractions.

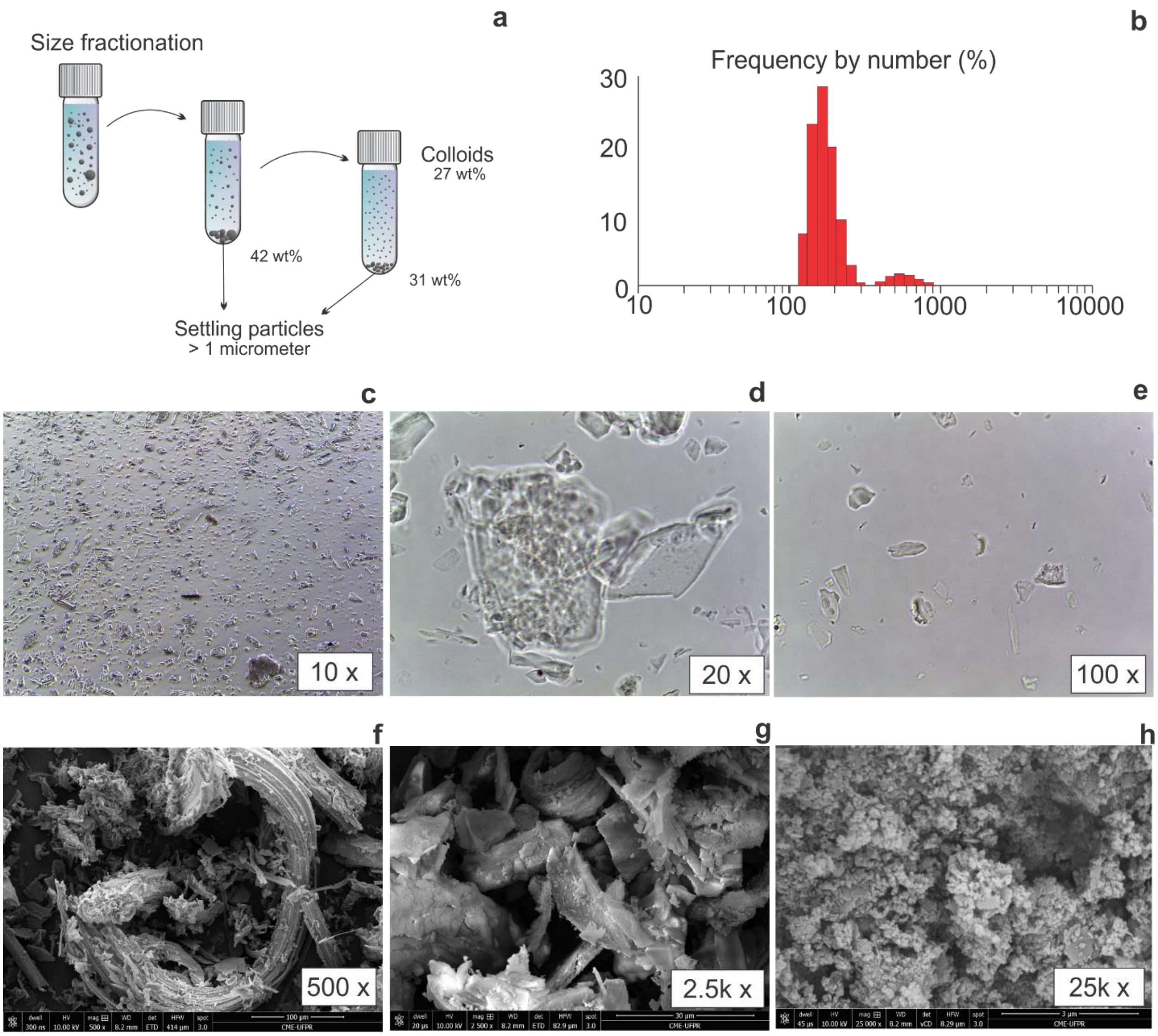

Fig. S2. Procedure adopted for size fractionation of biogenic silica (a). DLS results for the obtained colloidal fraction (b). Optical images of settling biogenic silica particles at 10 (c) and 20 (d) times of magnification showing their irregular shapes derived from cell wall plant. Optical image of the colloidal fraction of biogenic silica (c). Scanning electron microscopy images at 25k times of magnification (h) stating their high porosity even being not colloidally stable fractal-like particles (g,g).

Surface characterization of $BSiO_2$ nanoparticles through XPS analysis. The XPS analysis stating the surface functionalization is presented in the **Fig. S3**. The same characteristic carbon peak related to the C-H and C-C chemical structures was observed at 285 eV for the SiOH and $SiNH_2$ particles (**Fig. S3c**); however, a new carbon moiety with peak at 288.5 eV was verified after deconvolution of the high-resolution carbon spectra of the SiCOOH particles (**Fig. S3d**). The high-resolution XPS spectra of N_{1s} for the SiCOOH (**Fig. S3g**) particles shows notable differences when compared to the $SiNH_2$ (**Fig. S3f**). Free amino groups appear at 399.1 eV for both $SiNH_2$ and SiCOOH particles; however, the contribution of these groups to the total nitrogen response decreased from 63.7 to 32.6% after carboxyl-functionalization because part was converted into amide groups via ring opening linker elongation reaction with maleic anhydride. The interactions between neighboring amino groups undergo H-bonding or between amino groups and unreacted silica hydroxyl groups appeared at around 401.5 eV. Again, this peak had lower contribution (20.0%) in the N_{1s} main peak for the SiCOOH particles when compared to the $SiNH_2$ (36.3%). A small shift was observed for this component, and it may be attributed to the interaction of the amino groups with OH moieties from carboxyl termination instead with those from silanol groups. After deconvolution, a new nitrogen component related to the amide group appeared at 399.9 eV and it contributed to 47.5% of the total N_{1s} spectrum of the SiCOOH particles. From these results, it is possible to say that the free amino groups were partially converted into amide groups via chemical reaction with maleic anhydride resulting in the carboxyl termination.

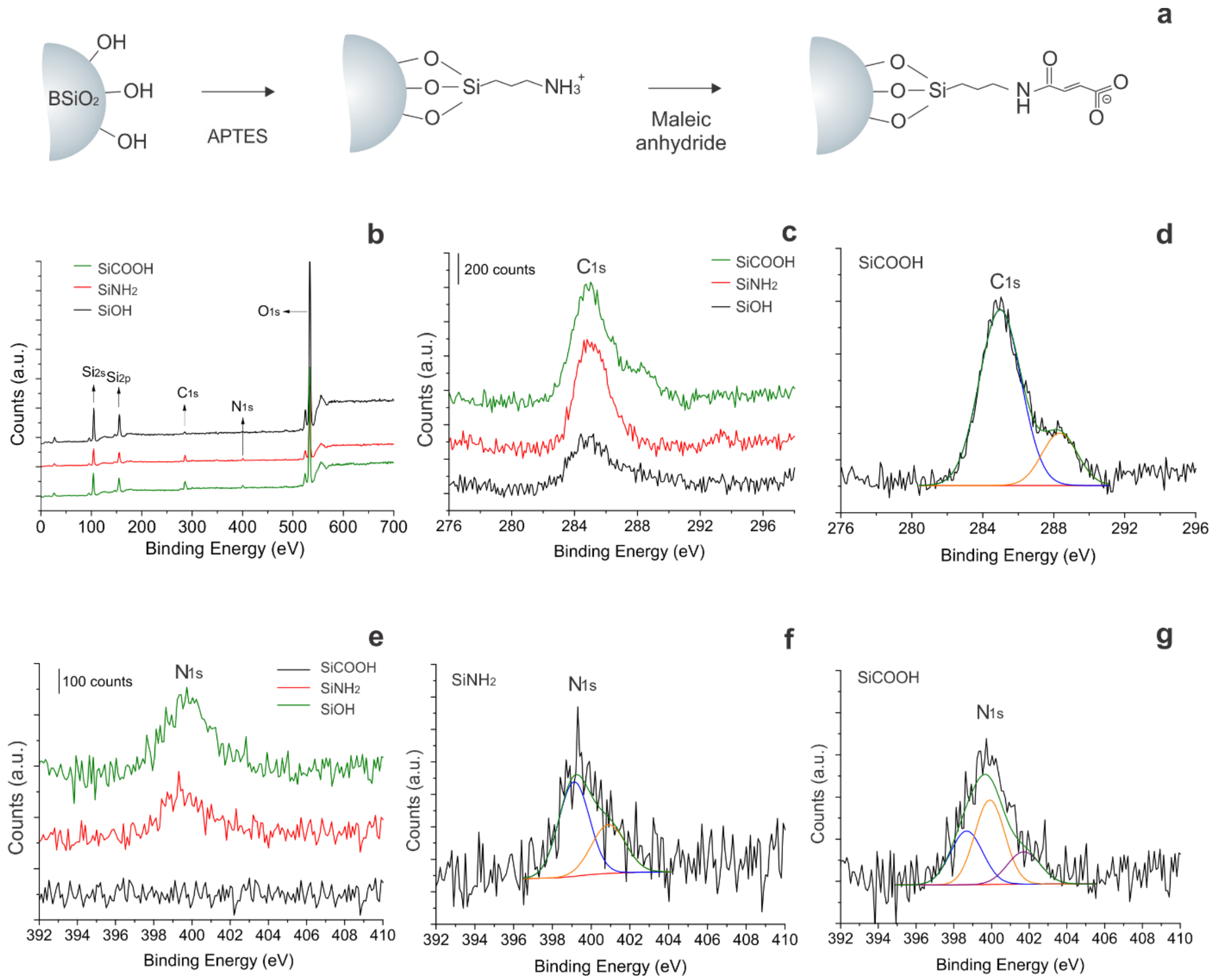

Fig. S3. Stepwise surface chemical modification of $BSiO_2$ (a). Survey XPS spectra of as-prepared and surface modified biogenic silica particles (b). Carbon C_{1s} high-resolution spectra (c) and deconvoluted version for SiCOOH particles (d). Nitrogen N_{1s} high-resolution spectra (e) and deconvoluted versions for $SiNH_2$ (f) and SiCOOH (g) particles.

Thermal decomposition patterns of biogenic silica.

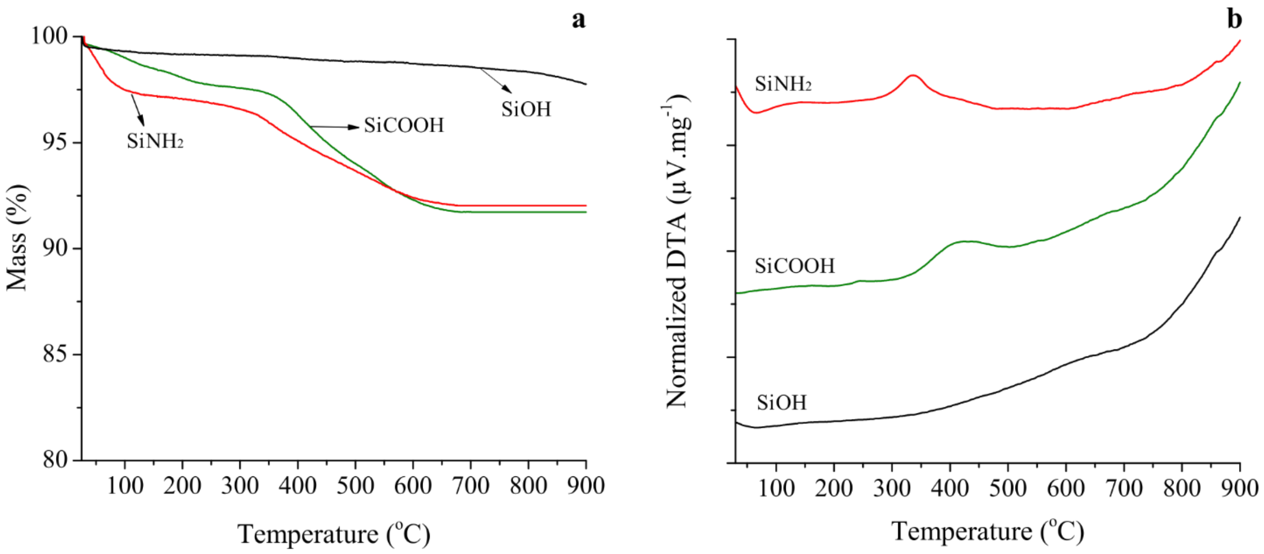

Fig. S4. Thermogravimetric (a) and thermal differential (b) curves of the as-prepared and functionalized biogenic silica particles.

Fourier-Transformed Infrared (FTIR) spectra of the functionalized particles.

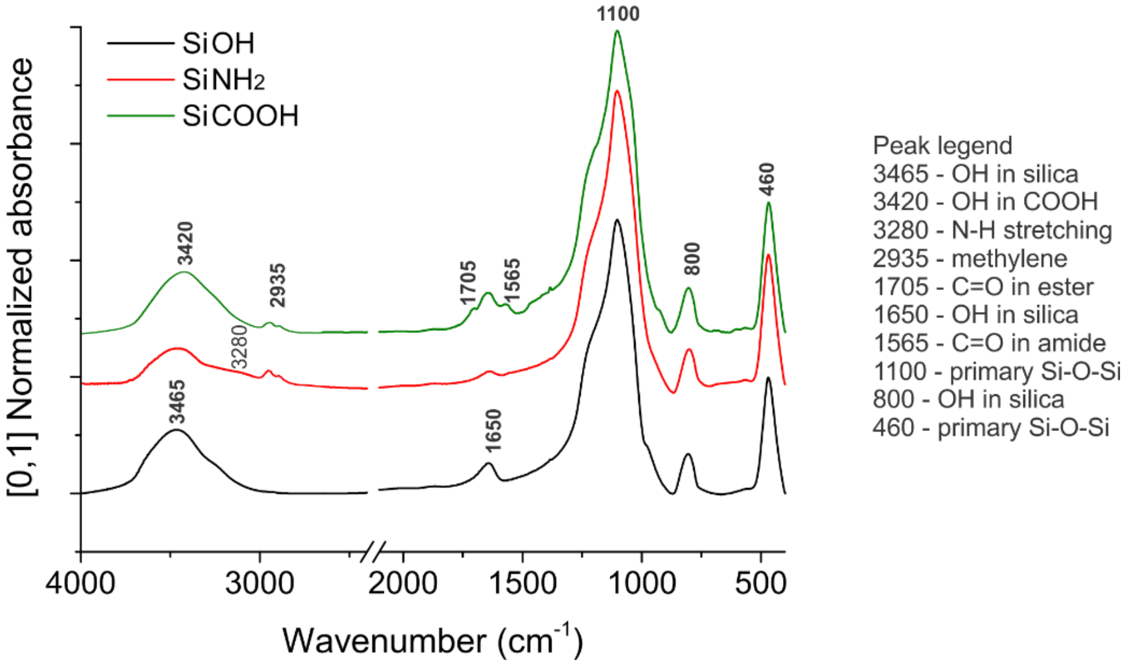

Fig. S5. FTIR spectra of the unmodified and modified biogenic silica particles detailing the assignments for each identified peak.

Extra information on the XPS results of the obtained BDS.

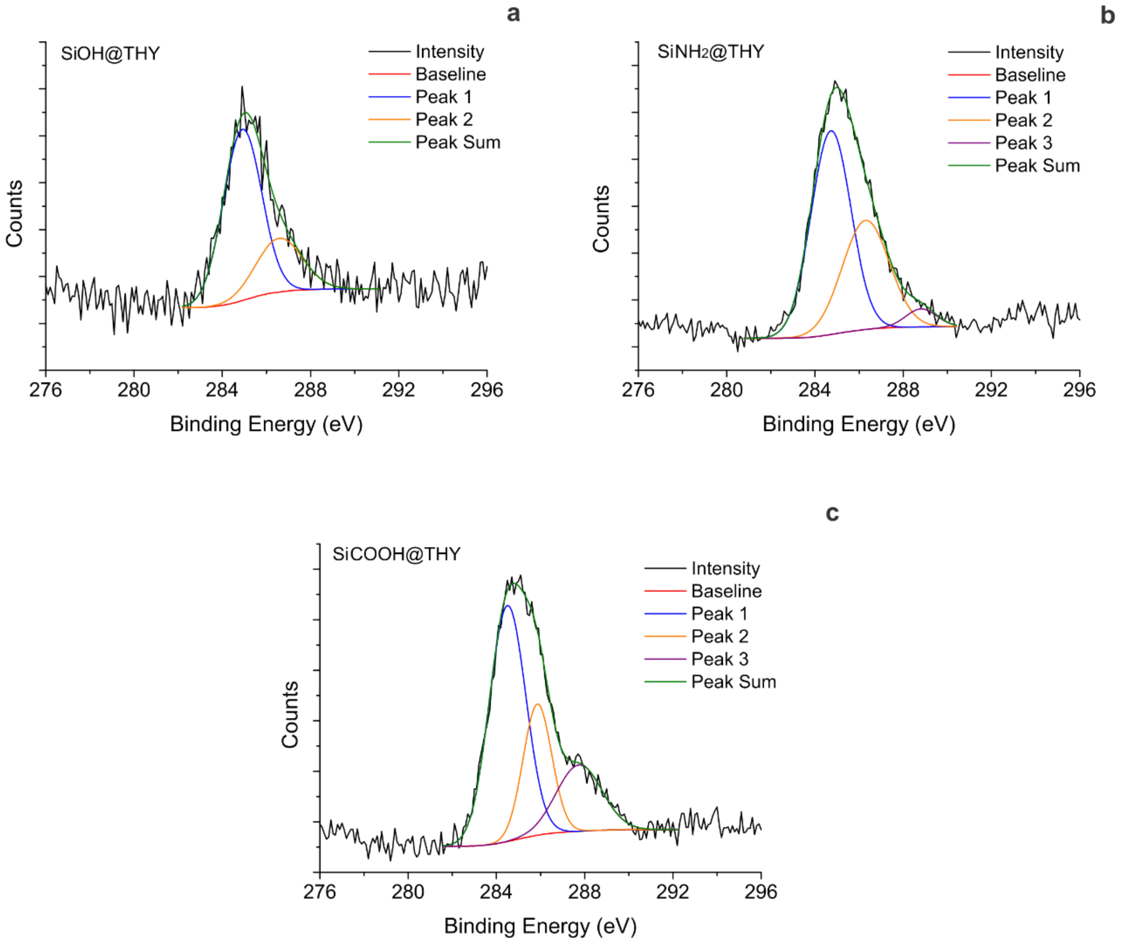

Fig. S6. Deconvolution of the C_{1s} high-resolution spectra of the SiOH@Thy (a) SiONH$_2$@Thy (c) SiCOOH@Thy (d).

Effect of size fraction on the thymol release profiles, fitting of kinetic models on the experimental thymol release profiles, theoretical release rate tuning via BDS ratio compositions, and activation energy calculation.

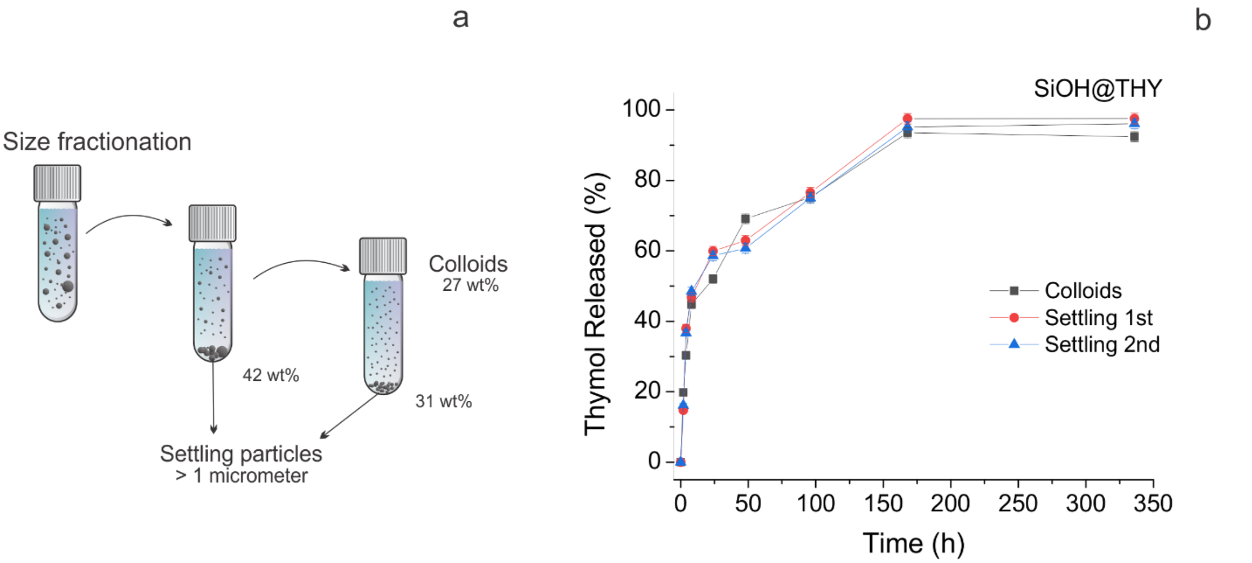

Fig. S7. Size fractionation procedure for obtaining three different sizes of bio-sourced silica (a). Thymol release rate from SiOH@Thy prepared with the different silica particle sizes showing that there is no influence of the particles sizes (in the studied range) on the delivery (b).

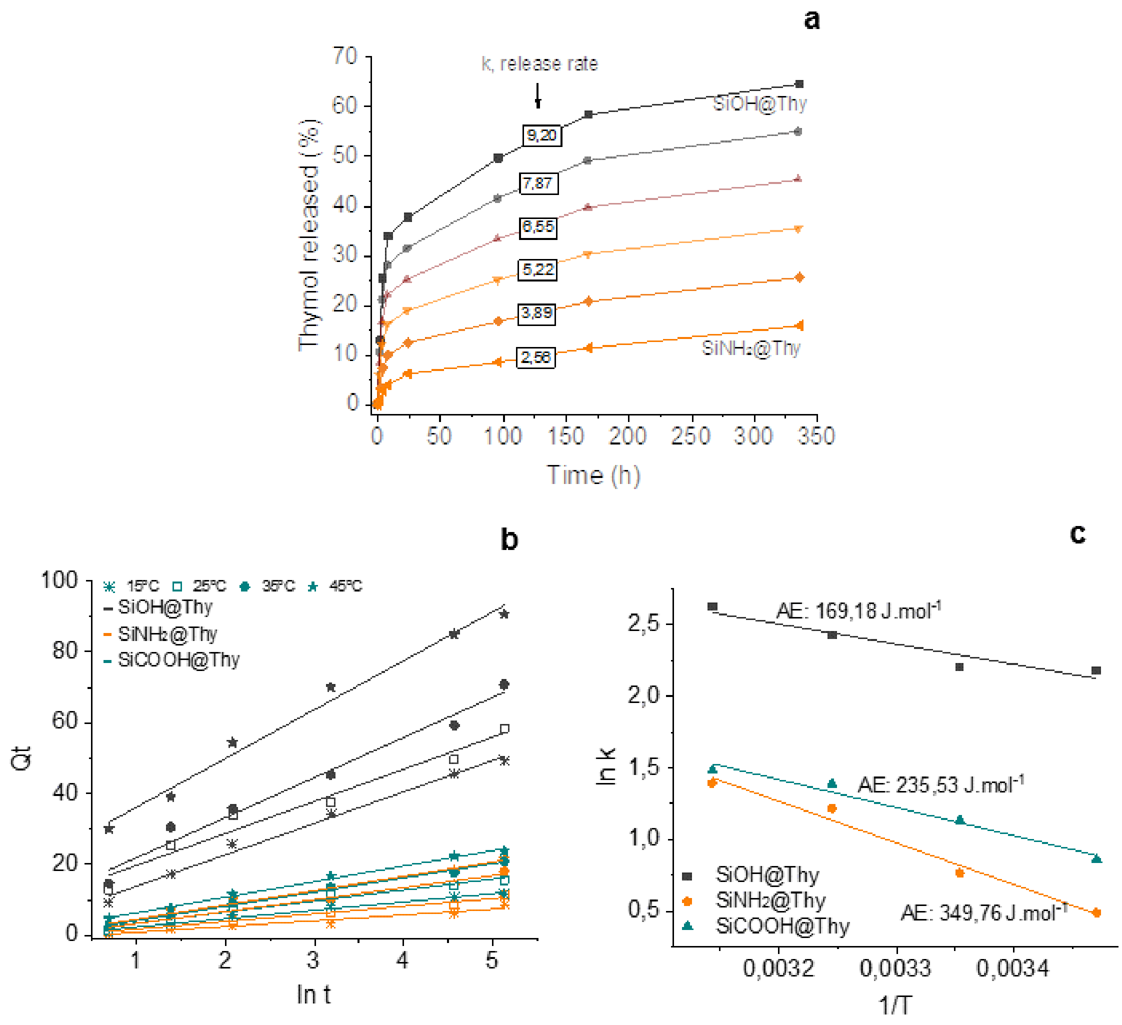

Fig. S8. Theoretical thymol profiles with tethered release rate through fraction compositions between the BDS with the fastest and slowest release rate (a). Elovich linearization of the thymol release out profiles at different temperatures for all obtained BDS (b) in order to calculate the activation energy of their release using the Arrhenius plot (c). An additional release profile at 15oC was carried out in order to precisely obtain the activation energy.

Control plates of the agar diffusion assay.

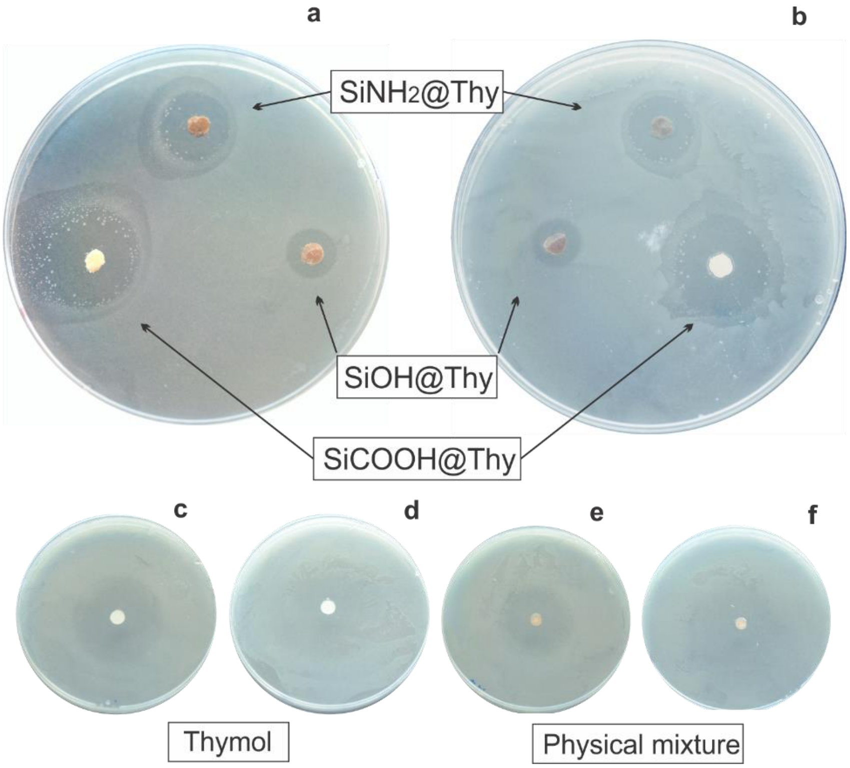

Fig. S9. Agar diffusion test results on *Escherichia coli* (a) and *Staphylococcus aureus* (b) plates treated with SiOH@Thy, $SiNH_2$@Thy and SiCOOH@Thy BDS discs. *Escherichia coli* plates treated with undissolved thymol (c) and mixed thymol/silica discs (e). *Staphylococcus aureus* plates treated with undissolved thymol (d) and mixed thymol/silica discs (f).

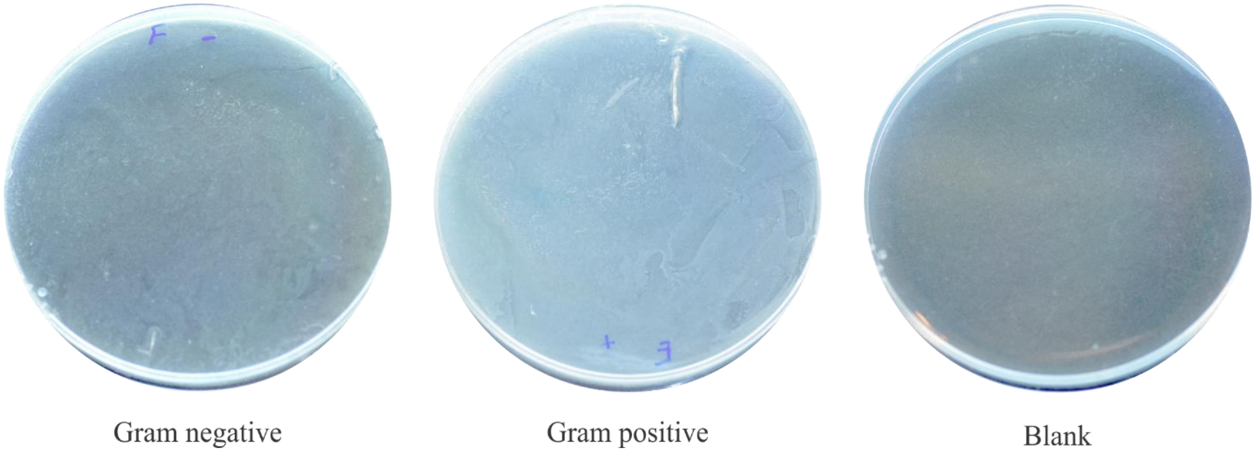

Fig. S10. Bacteria plates used as internal controls for the agar diffusion assay. Left: *Escherichia coli* plate with no treatments. Center: *Staphylococcus aureus* plates with no treatments. Right: Mueller-Hinton agar plates without any cell.

Table S1. Analysis of variance and least significant difference (LSD) test to evaluate the thymol loading as a function of the silica surface

Factor	Sum of squares	df	Mean Squares	F-ratio	P value
Between	170.586	2	85.2929	5.65	0.0521
Within	75.4701	5	15.094		
Total	246.056	7			

Sample	Mean (mg/g)	LSD* Groups
SiOH@Thy	73.65	b
$SiNH_2$@THY	61.78	a
SiCOOH@Thy	65.58	ab

* Different letters along the same column indicates statistical difference at 5% of probability of error.

Table S2. Analysis of variance and least significant difference (LSD) test to evaluate the total biocide release as a function of the silica surface

Factor	Sum of squares	df	Mean Squares	F-ratio	P value
Between	4569.45	2	2284.72	115.41	0
Within	118.779	6	19.7965		
Total	4688.22	8			

Sample	Mean (%)	LSD* Groups
SiOH@Thy	64.62	b
$SiNH_2$@THY	17.77	a
SiCOOH@Thy	15.92	a

* Different letters along the same column indicates statistical difference at 1% of probability of error.

Table S3. Coefficient of determination of the kinetic models applied as a tentative to explain the release of thymol out from biogenic silica

Model	Equation	SiOH@Thy	$SiNH_2$@Thy	SiCOOH@Thy
Zero Order	$Qt = kt$	0.78	0.67	0.86
First Order	$Qt = e^{-kt}$	0.57	0.43	0.60
Second Order	$1/Qt = kt$	0.37	0.23	0.34
Parabolic-diffusion	$Qt = kt^{1/2}$	0.88	0.81	0.94
Korsmeyer-Peppas	$\log Qt = \log k + n \log t$	0.78	0.85	0.91
Elovich	$Qt = k\ln t$	0.96	0.96	0.98

Qt = amount released at time *t*; *t* = time; *k* = release rate constant; *n* = kinetic exponent of the Korsmeyer-Peppas model.

* The equations were presented in their integrated forms and considering an initial 0 concentration of the biocide in the release microenvironment.

Table S4. Multifactorial analysis of variance and least significant difference test (LSD) to evaluate *k* over temperature changes as a function of the silica surface

Factors	Sum of squares	df	Mean Squares	F-ratio	P value
Surface	368.061	2	184.03	174.87	0
Temperature	33.5608	2	16.7804	15.94	0.0001
Residual	23.1528	22	1.0524		
Total	424.775	26			

Sample	Mean (Qt/e^t) and LSD groups*		
	25 ^oC	35 ^oC	45 ^oC
SiOH@Thy	9.15 b A	10.86 b A	14.34 b B
$SiNH_2$@Thy	2.62 a A	3.60 a B	4.17 a B
SiCOOH@Thy	3.01 a A	3.9 a B	4.43 a B

* Different letters (lowercase along the same column, and uppercase along the same row) indicate statistical difference at 1% of probability of error.

Table S5. Multifactorial analysis of variance and least significant difference test (LSD) to evaluate *k* over pH changes as a function of the silica surface

Factors	Sum of squares	df	Mean Squares	F-ratio	P value
Surface	235.124	2	117.562	50.98	0
pH	29.9066	2	14.9533	6.49	0.0061
Residual	50.7281	22	2.30582		
Total	315.759	26			

	Mean (Qt/e^t) and LSD groups*		
Sample	pH 5	pH 7	pH 9
SiOH@Thy	12.33 c B	9.15 b A	8.82 c A
$SiNH_2$@Thy	2.16 a A	2.62 a B	4.77 b C
SiCOOH@Thy	7.85 b B	3.01 a A	3.58 a A

* Different letters (lowercase along the same column, and uppercase along the same row) indicate statistical difference at 1% of probability of error.

Table S6. Multifactorial analysis of variance and least significant difference test (LSD) to evaluate *k* over salinity changes as a function of the silica surface

Factors	Sum of squares	df	Mean Squares	F-ratio	P value
Surface	300.02	2	150.01	101.61	0
Temperature	70.1809	2	35.0905	23.77	0
Residual	32.4806	22	1.47639		
Total	402.682	26			

Sample	Mean (Qt/e^t) and LSD groups*		
	0.5%	2.0%	3.5%
SiOH@Thy	9.15 b A	10.36 c A	15.45 c B
$SiNH_2$@Thy	2.62 a A	5.86 b B	6.24 b B
SiCOOH@Thy	3.01 a A	4.94 a B	4.92 a B

* Different letters (lowercase along the same column, and uppercase along the same row) indicate statistical difference at 1% of probability of error.

Table S7. Multifactorial analysis of variance and least significant difference test (LSD) to evaluate the bacterial growth of each bacteria culture treated with different samples

Factors	Sum of squares	df	Mean Squares	F-ratio	P value
Sample	0.009983	4	0.002496	10.58	0
Bacteria	0.003099	1	0.003099	13.14	0.0014
Residual	0.005662	24	0.000236		
Total	0.018744	29			

	Mean (a.u.) and LSD groups*	
Sample	Gram -	Gram +
SiOH@Thy	1.079 b A	1.083 c A
$SiNH_2$@Thy	1.106 c A	1.074 c A
SiCOOH@Thy	1.113 c B	1.066 bc A
Thymol	1.060 ab B	1.033 a A
Physical Mixture	1.054 a A	1.054 b A

* Different letters (lowercase along the same column, and uppercase along the same row) indicate statistical difference at 1% of probability of error
